# Compressed Air in Dentistry

**Published:** 1903-03-15

**Authors:** S. E. Knowles

**Affiliations:** San Francisco


### COMPRESSED AIR IN DENTISTRY.

BY S. E. KNOWLES, M.D., D.D.S., SAN FRANCISCO.

Read before the California State Dental Association, June, 1902.

In selecting this title for an essay, the beaten path has been somewhat departed from and an effort made to present a subject which, while not entirely new, will, it is hoped, prove to be novel in its application in some particulars. The only apparatus for the purpose made by any of the manufacturers of dental appliances, so far as my knowledge extends, is the one made by the S. S. White Dental Manufacturing Company, which has been upon the market ten or twelve years and which consisted of a tube of black vulcanite with a rubber bulb upon one end, and upon the other a glass tube curved and drawn to a point, inside of which was a coil of small platinum wire connected with the 110-volt direct-electric current, and was operated by compressing the bulb in the hand to produce the current of air. This apparatus was purchased and an effort made to use it, but almost at once two serious faults developed. The first, which was afterward rectified, was the frequent breaking of the glass from contact with the red-hot platinum wire. This was overcome by rolling asbestos paper around a single strand of the platinum wire and winding the wire back and around the paper to the point of beginning. The second fault was the uneven application of the air to the tooth, because of the surging of the current of air, inseparable from the working of the small bulb. In the action of this implement the air would be first too cold, then too hot, and at the end of the impulse again too cold and was the cause of frequent protests on the part of the victim. This suggested the wisdom of adopting some device to maintain a regular, even flow of the air. The force bellows belonging to a Fletcher's blast melting furnace was then substituted for the bulb and attached, after cutting off the bulb. This bellows was worked by the foot of an assistant and as it gave a pressure of five or six pounds was a decided improvement, although the uneven pressure was still present in a mitigated form. This was used for several years and gave moderately good results, but was far from perfect, unsightly and inconvenient. The next step was to arrange for the storage of air in quantity under greater pressure. To this end a hand compressor, which was worked with a wood-sawing motion, with reservoir of a capacity of about five gallons, was purchased, but the capacity of the tank was much too little, so much so, that a pressure of fifty pounds lasted but a few minutes, and, as the labor of compressing the air was considerable, it was decided to add the latest acquisition to the junk pile. This decision was in a measure brought about by my assistant modestly suggesting that proficiency in sawing wood was not absolutely essential as a part of his dental education. This brought us to the point of considering some appliance operated by some power other than human. The various machines for utilizing water power were inspected and passed as not being quite the thing, and eventually it was decided to give the electric motor a trial. The one used at the chair is of one-sixth of one horse power, quite ample for the purpose. The dentist is proverbially ingenious. During my spare hours an air pump was made of brass and attached to the motor. The pump compressed one cubic inch of air at each revolution of the armature and would give a pressure of sixty pounds an hour after starting the pump. It was found, however, that so much heat was generated that the addition of a water jacket was necessary to keep the lubricating oil from burning. The air reservoir used was an ordinary hot water tank, of thirty gallons capacity, such as is used in connection with the kitchen stove. This tank would stand one hundred and fifty pounds pressure. While this apparatus was in use and giving satisfaction, a circular was received from the manufacturer of an electric air pump which was so promising that it was determined to give it a trial. Accordingly, through one of the local dental goods dealers, an order was given and the pump procured and installed. The pump was so constructed as to automatically maintain any desired pressure up to fifty pounds, the pressure of air operating a switch in such a manner as to keep the air pressure between a maximum and minimum, to be determined at will. The one in use was set for pressure between twenty and thirty pounds, that pressure having been found by experience to be quite satisfactory. Block tin pipe of one-eighth and one-quarter of an inch caliber was used to carry the air to the chairs and the laboratory bench. Having thus roughly described the history of the plant we will pass at once to the various uses to which it may be put by the dentist.

In the laboratory for soldering, melting metals in the Fletcher's furnace, annealing and similar operations. The blast is much more easily governed than by using the foot bellows, as the surging of the air pressure is entirely absent. The pressure when turned on is more than sufficient for the full head of gas, and makes an apparatus absolutely without fault.

At the chair there are several uses of minor importance, such as operating sprays of the various medicines used about the mouth; testing suspected teeth with hot and cold air under the dam, and other similar uses which no doubt would readily suggest themselves to you, but in my hands the principal use has been in the treatment of pulpless teeth. It is my firm belief that the hot current of air has given results in sterilizing the lifeless dentine that can not be even approached by any other method with which I am acquainted. Permit me to digress in order to make the point perfectly clear. After the pulp is dead, all of the organic matter in the dentine is cut off from nourishment, dies and becomes a menace to the safety of the tooth. About all the treatment usually applied by the dentist is directed to the removal of the debris of the dead and frequently putrescent pulp, sterilizing the canals and chamber and filling the whole recess in the tooth, canals, chamber and cavity, practically paying no attention to the decaying organic matter mingled with the lime in the dentine. Did you ever consider what will be the result most likely to follow this condition of things? Is it surprising that eventually the organic matter in the dentine degenerates, forming infectious fluids or gases or both, causes the death of the cementum and pericemental membrane and invites the expulsion of the tooth through ulceration? When one recommends immediate canal filling after the pulp has been killed, or in case of spontaneous death of the pulp as soon as the canal has been rendered sterile, it is fair to conclude that the situation is not fully understood. In order that the dentine may be rendered fully sterile, the hot air drying must be persisted in, until the moisture is fully expelled from the dentine, clearing the way for the sterilizing fluids, which are often of oily nature, as in case of my favorite agent, creosote. Inasmuch as it is a very rare thing for me to kill a pulp, most of the cases treated involve putrescent canals, some of which have been in such condition as to render the term putrescent wholly inadequate to describe the situation. The detailed description of the treatment is as follows: If the chamber is without vent, at the first sitting, the dam having been applied, the vent is made, the cavity opened so as to give free access to the pulp chamber, the debris removed and creasote on cotton introduced, into the chamber; upon this is placed a loosely rolled piece of cotton thus permitting the escape of gas and fluid into the mouth. Care is taken not to interfere in any manner with the canals, either by use of the probe or by pressing upon the cotton as the septic matter may be thus forced through the apices of the canals and arouse the slumbering volcano. Upon the second or third day the tooth may be thoroughly explored while under the dam, canals included, without special danger. After cleaning as thoroughly as possible the heated air is used, blanching the dentine, the creosote or other agent may now be introduced, led into the canals with soft plain broaches and the air applied again until the sterilizing fluid is absorbed by the porous dentine, a piece of cotton saturated with the sterilizing agent is left in the pulp chamber and cotton and shellac or the gutta percha cavity seal inserted to keep out foreign substances. Once or twice a week the creosote on cotton is renewed and continued in this manner for several weeks until the cavity will stand perfect occlusion, without evidence of pericemental tenderness, when an appointment may be given for permanently sealing the canals and inserting the filling. In the operation of filling, the dam is placed in position, the stain on the cavity walls and pulp chamber removed, the canals enlarged and straightened and the shaping of the cavity completed, the hot air again thoroughly applied, creosote again introduced and driven into the dentine, cones of gutta percha dipped in creosote placed in the canals and pressed into their places with cold points, after heating the cones with the hot air. This method prevents the cones from sticking to the canal filling instruments. The pulp chamber is now filled with artificial bone, after the removal of the surplus creosote with cotton or other drying material. If an amalgam is to be used in the tooth, it may be applied at once without danger, but if gold is to be used, by all means defer its insertion at this time, and use a temporary plastic filling in the cavity, allowing the tooth to rest for at least six months, as the effect of malleting the tooth at this time would be dangerous. It has been my experience that a tooth treated in this manner is as safe as it is possible to make a pulpless tooth.

### DISCUSSION.

Dr. Deichmiller. Mr. President, Ladies and Gentlemen: I will not take up your time with eulogistic remarks further than to say that Dr. Knowles deserves much praise and commendation for his excellent paper, and for his patient and painstaking persistence in perfecting his apparatus for the use of hot air. I have used it for a number of years in my practice and everyone who has aroused the sleeping volcano in the shape of a blind abscess will testify to the fact that anything that will reduce the frequency with which these blind abscesses appear is welcome in dentistry. Dr. Knowles spoke of the difficulty in adapting this to his practice. I have been rather fortunate in having been associated with a specialist and occulist, who had a tank which I have been permitted to hitch on to with my lead pipe carrying air at a compression of 35 pounds to my office. I immediately procured an apparatus to heat the air as it was forced from this tank to the hand-piece or cut-off, as it is called.

You have no idea of the comfort I have had in the use of hot air. Many uses will suggest themselves as one goes along. I believe Dr. Knowles so far has adapted it principally to the drying out of septic root canals. I use it in the laboratory for melting metals and for the furnace and the blow-pipe and many other ways in the laboratory that will suggest themselves.

I have jotted down a few here that I may be more correct in my statement and take up less of your time.

For instance in the sterilizing of the mouth before performing surgical operations. When a patient applies to you with an alveolar abscess and possibly pyorrhea alveolaris, his mouth is usually in a very septic condition. To plunge a knife into this abscess and then with a drill to drill or curette the bone without sterilizing the mouth it seems to me is a very dangerous procedure, because you will carry infection with it from the surface of the mucous membrane unless you sterilize the mouth thoroughly. That can be done with the spray bottle attached to the cut-off with a solution of say 1 1-2 to even 3 percent pyrozone, and a few drops, say 15 drops of listerine or any of the antiseptics that are used at the chair, and you have an efficient sterilizer there that will, in great measure at least, accomplish the purpose. In such cases I find it a protection to polish the teeth with pumice before proceeding, then sterilize the mouth afterwards, then go on with the opening of the abscess. In that way I feel confident that I am taking at least the minimum amount of infection into the field of operation.

In reducing retching from the rubber dam. We have patients many times who retch when the rubber dam is being used. Or before taking impressions, the same thing occurs. This may be accomplished by spraying a 2 percent solution of cocaine into the nostril.

Sometimes in regulating, the ligatures will work up and inflame the gingiva. After the ligatures are removed simply direct a spray of pyrozone and the inflammation will soon disappear.

For inflammation generally involving the gums, the inner surface of the cheek, and the oral cavity in general, the treatment of pyorrhea alveolaris and all septic conditions of the oral cavity, the spray is much better, I think, than the syringe point. Of course with this platinum point syringe, you can sterilize thoroughly by passing through a gas or alcohol flame, but where delicate granulations begin to form, you disturb them very much with the pointed syringe, and the healing process will be delayed, but with the spray, with the force of 30 pounds upon the supply of air, the medicament can be forced between the gums, in the apex of the tooth, and everywhere, without danger involved by a pointed instrument.

The treatment of the antrum can be facilitated by this spray and the nasal passages where the antrum has become infiltrated and inflamed, by extension to the nasal passages, can be readily reached with the spray.

For the cleansing or sterilizing of the alveolæ after extraction of an abscessed tooth, or after curetting the socket, or before replantation or implantation, cleansing either the artificial alveolus or the natural one, the spray is very beneficial.

A stream of cold air reduces pain in periostitis.

You, that have had patients come with severe pericementosis in which the tooth is lifted out of its socket, have no doubt had your ingenuity tested how to get in and relieve the pressure and allow the escape of gas without inflicting great pain. If you will direct a stream of cold air upon that you can open up the canal and allow the escape of gas from the putrescent contents of the pulp canal with very little pain, and sometimes it is reduced to such a minimum that the patient scarcely feels it.

Dessicating the tooth, obtunding sensitive dentine, is another use.

In drying the tooth before fillings are inserted, I use a preparation of chloroform, carbolic acid and a little alcohol that rapidly vaporizes and sterilizes. If a gutta percha filling is to be used just keep directing a stream of hot air on the tooth, and the gutta percha will directly adhere to the warm surface without adhering to the instrument. Bleaching discolored teeth is another use for the hot air.

I heat up a tooth in which the pulp has been destroyed so that the gutta percha will begin to soften, then with a continual stream of hot air I can put it into place without it adhering to my pulp canal instrument.

To force cement into root canals when setting a crown is another important use of compressed air. If you have never tried it you will find a great deal of comfort from this method.

To reduce the heat while excavating cavities with the bur particularly in cutting enamel with cross-cut fissure burs.

Also while using paper discs and strips, I have found a great deal of comfort, as well as the work being very much expedited by the use of cold air upon sandpaper discs, as well as a little vaseline.

Reducing the heat created in trimming teeth for crowns is another important use.

Another very important use is hardening modeling compound by a stream of cold air.

Then I believe the doctor has spoken of thermal tests for dead teeth—for instance, a lower molar in which the anterior pulp is alive and the posterior one is dead. It is a good means of diagnosis.—*Pacific Gazette.*

